# Factors Influencing the Proportion of Non-examinees in the Fukushima Health Management Survey for Childhood and Adolescent Thyroid Cancer: Results From the Baseline Survey

**DOI:** 10.2188/jea.JE20180247

**Published:** 2020-07-05

**Authors:** Kunihiko Takahashi, Hideto Takahashi, Tomoki Nakaya, Seiji Yasumura, Tetsuya Ohira, Hitoshi Ohto, Akira Ohtsuru, Sanae Midorikawa, Shinichi Suzuki, Hiroki Shimura, Shunichi Yamashita, Koichi Tanigawa, Kenji Kamiya

**Affiliations:** 1 Department of Biostatistics, Nagoya University Graduate School of Medicine, Aichi, Japan; 2 National Institute of Public Health, Saitama, Japan; 3 Radiation Medical Science Center for the Fukushima Health Management Survey, Fukushima Medical University, Fukushima, Japan; 4 Graduate School of Environmental Studies, Tohoku University, Miyagi, Japan; 5 Department of Public Health, Fukushima Medical University School of Medicine, Fukushima, Japan; 6 Department of Epidemiology, Fukushima Medical University School of Medicine, Fukushima, Japan; 7 Department of Radiation Health Management, Fukushima Medical University School of Medicine, Fukushima, Japan; 8 Department of Thyroid and Endocrinology, Fukushima Medical University, Fukushima, Japan; 9 Department of Laboratory Medicine, Fukushima Medical University School of Medicine, Fukushima, Japan; 10 Fukushima Medical University, Fukushima, Japan; 11 Nagasaki University, Nagasaki, Japan; 12 Center for Advanced Radiation Emergency Medicine, National Research Institutes for Quantum and Radiological Science and Technology, Chiba, Japan; 13 Research Institute for Radiation Biology and Medicine, Hiroshima University, Hiroshima, Japan

**Keywords:** Fukushima Health Management Survey, thyroid cancer screening, participation rate, non-examinees, logistic regression

## Abstract

**Background:**

After the Fukushima Daiichi Nuclear Power Plant accident, a preliminary ultrasound-based screening for thyroid cancer was conducted to establish a baseline for subsequent evaluations. In this survey, we assessed the relationship between the proportion of non-examinees and characteristics of the target populations.

**Methods:**

After summarizing a regional difference of non-examinees among the population of 359,200 (primary evaluation) and 2,246 (confirmatory testing) individuals who were living in Fukushima Prefecture on March 11, 2011, we estimated odds ratios (ORs) for each characteristic, including age, sex, area of residence, and moving after the accident, based on the proportion of non-examinees for the primary examination and the confirmatory testing, using a multivariate logistic regression model.

**Results:**

The dataset included 64,117 non-examinees (primary evaluation) and 194 (confirmatory testing). The logistic regression result indicated that girls were not likely to be non-examinees compared to boys, with adjusted OR of 0.80 (95% confidence interval [CI], 0.78–0.81) for the primary evaluation. Odds were lowest for children 6–10 years old (OR 0.26; 95% CI, 0.25–0.27), and higher for those 11–15 years old (OR 1.28; 95% CI, 1.25–1.32) and over 16 years old (OR 5.30; 95% CI, 5.16–5.43) when compared to children 0–5 years old. Individuals residing in the western part of the prefecture showed higher ORs. There was a higher proportion of non-examinees among those who moved after the accident compared to those who did not in the primary evaluation (OR 1.72; 95% CI, 1.64–1.79).

**Conclusions:**

In addition to demographic characteristics, a change of residence could be a potential factor that influenced the proportion of non-examinees. Our results will help proper interpretation of reports and prospective management of the survey.

## INTRODUCTION

After the nuclear power plant accident caused by the Great East Japan Earthquake on March 11, 2011, the Fukushima Health Management Survey^1^ (FHMS) was conducted to support the long-term health of Fukushima residents. In October 2011, as a part of the FHMS, the Fukushima prefectural government started an ultrasound-based screening program for thyroid cancer (thyroid ultrasound examination [TUE]) for all residents aged 18 years or younger who were living in Fukushima Prefecture on the day of the Fukushima Daiichi Nuclear Power Plant accident. The baseline survey of TUE (also referred to as the first round of TUE, or preliminary baseline screening) was conducted from October 2011 to April 2015 to establish a baseline for subsequent full-scale screening programs for evaluation in the same target population in a cohort study scheme.^1^ This screening program comprised a primary evaluation using ultrasound and a following confirmatory testing.^2^ Out of 300,473 children and adolescents who were examined, 116 (39 boys and 77 girls) were diagnosed with confirmed or suspected thyroid cancer (hereafter, thyroid cancer cases).^3^ This larger-than-expected number of cases incited the suspicion of an “epidemic” of radiation-induced cancer. Multiple reports, including the UNSCEAR report,^4^ have since discussed whether the number of observed cases was excessive. Tsuda et al^5^ have suggested that the geographical excess was introduced due to exposure of the residents to radioactive contamination in the environment. On the other hand, Ohira et al^6^ have concluded that there was no significant association between the prevalence of thyroid cancer cases and local radiation exposure levels. Furthermore, Katanoda et al^7^ have suggested a potential over-diagnosis of thyroid cancer; Takahashi et al^8^ and Midorikawa et al^9^ have considered the cancer-progression model related to the survey, and Nakaya et al^10^ have found no significant spatial anomalies/clusters or geographical trends of thyroid cancer prevalence among the ultrasound examinees.

For this kind of survey, as also pointed out in the above studies, the participation rate (ie, the proportion of examinees) is an important factor to be considered when interpreting the results. For instance, in order to investigate the influence of radiation on the regional distribution of observed thyroid cancer cases in TUE, the participation rate perhaps becomes a confounding factor for the estimation of the number of cases. The summary of results of the baseline survey of TUE, officially reported on March 31, 2017^11^ (hereafter, FHMS Official Report), showed that the participation rate was 81.7% for the primary evaluation and 92.9% for the confirmatory testing of the target populations. Although the rates in each municipality were also reported in the FHMS Official Report, not only the differences in participation, but also the demographic characteristics, should be clarified to assess the potential impacts of the number of thyroid cancer cases on the statistical analysis.

In this study, we assessed the relationship between the proportion of non-examinees in the baseline survey of TUE and the characteristics of individuals living in Fukushima Prefecture on March 11, 2011. In particular, we examined the differences in the odds of non-examinees between residence areas, and the influence of changing residences (moving) after the Great East Japan Earthquake.

## METHODS

### Materials

A total of 367,649 children and adolescents were registered for the baseline survey of TUE. The Fukushima Medical University, commissioned by Fukushima Prefecture, executed an ultrasound screening program and compiled the surveillance database, which formed the data source for this study. The database included sex, age, and the municipalities (city, town, and village) of residence for each individual on March 11, 2011, based on the resident registration. The municipalities of residence on March 11, 2011 and current residence were updated using spontaneous reports from the registered individuals. We analyzed the dataset that, as of October 25, 2017, contained 359,200 individuals living in Fukushima Prefecture on March 11, 2011 (97.7% of the total population in FHMS Official reports). Of those, 295,083 underwent the primary evaluation and agreed to provide information about the examination for this research. Information, such as date and diagnosis, were recorded in the dataset. Individuals without recorded information in the dataset were defined as non-examinees. Of note, the reports on the examination were not recorded in the dataset of this study for individuals without confirmed diagnosis for each examination or for those that did not agree to provide their information; thus, these individuals were analyzed as non-examinees in our study (eFigure 1). Therefore, following the primary evaluation and confirmatory testing, we finally identified 115 confirmed or suspected thyroid cancer cases in this dataset.

This study was approved by the Ethics Committee of the Fukushima Medical University (approval no. 1318). The Radiation Medical Science Center of Fukushima Medical University authorized us to analyze thyroid examination data from the FHMS. Data usage is currently restricted to the members or observers of expert committees of the FHMS. The members of the implementation headquarters committee of Radiation Medical Science Center for the FHMS were appointed by the Director of the Center of Fukushima Medical University. The members of each expert committee of internal and external organizations were approved by the implementation headquarters committee.

### Examination procedure

In the primary evaluation with thyroid ultrasound, the following diagnostic criteria were used: A, small or no nodule/cyst; B, further examination is necessary; and C, urgent need for further examination. For persons diagnosed as B or C in the primary evaluation, confirmatory testing was performed at the Fukushima Medical University Hospital or another hospital (certified by the expert committee) for advanced ultrasound examination. The confirmatory testing was performed at institutions that employ suitably qualified examiners or pathologists. Details of the survey protocol are provided elsewhere.^1^^,^^2^

The municipalities chosen for the primary evaluation were allocated by fiscal years: 2011 for the evacuation zone comprising 13 municipalities near the Fukushima Daiichi Nuclear Power Plant, 2012 for 12 municipalities in the middle part of the prefecture, and 2013 for the remaining regions (34 municipalities).^12^ Notification for each examination was sent to each person by mail.

### Statistical analysis

For the primary evaluation and confirmatory testing, we first calculated the proportion of non-examinees among the target population in each municipality of residence on March 11, 2011. For a supplementary analysis, we applied a cluster detection test using flexible scan statistic^13^^,^^14^ to investigate a possible regional difference in the proportion of non-examinees (see eAppendix 1).

**Figure 1.**
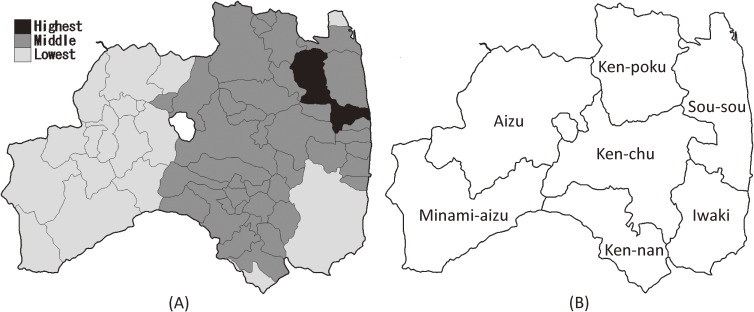
Two types of districts from 59 municipalities in the Fukushima Prefecture: (A) three areas based on the estimated degrees of exposure to radiation (modification of Figure 1 from Ohira (2016)^7^), (B) seven areas based on the administrative districts.

Next, we estimated the adjusted odds ratio (OR) for each characteristic based on the proportion of the non-examinees estimated from the primary evaluation and confirmatory testing using multivariate logistic regression models. We considered four factors, including age, area of residence on March 11, 2011, sex, and moving after the Great East Japan Earthquake. Among the 59 municipalities, two types of districts were examined for the area of residence. The first included three areas based on the estimated degrees of exposure to radiation based on the Basic Survey of the FHMS^7^: highest dose area (≥1% of the municipality received an external radiation exposure of ≥5 mSv; 2 municipalities), middle dose area (<1% of the municipality received ≥5 mSv, <99.9% received ≤1 mSv; 39 municipalities), and lowest dose area (≥99.9% received ≤1 mSv: 18 municipalities) (Figure 1A). The second type of districts included seven areas based on the administrative structure: Ken-poku (8 municipalities), Ken-chu (12 municipalities), Ken-nan (9 municipalities), Aizu (13 municipalities), Minami-aizu (4 municipalities), Sou-sou (12 municipalities), and Iwaki (1 municipality) (Figure 1B). Categories for age (0–5, 6–10, 11–15, and over 16 years old) and sex (boys and girls) were defined as in the FHMS Official Report. The moving status was classified as staying (remaining), moved, and unknown based on the change in the municipalities of residence between March 11, 2011 and the current recorded address in the dataset. The individuals whose information of current residence was missing were defined as unknown. Interaction terms of area and moving status were also evaluated in the model, and the best subset of interaction terms was selected by a stepwise method based on the Akaike’s information criterion (AIC).^15^ In addition, we predicted the probability *q_i_* for being a non-examinee for each individual *i* from the logistic regression model, and we determined the distribution of the *q_i_* for each of the target populations, the examinees in the primary evaluation, and the 115 confirmed or suspected thyroid cancer cases. As an application of the predicted values, a simple weighted estimation of the number of cases was shown in consideration of the probabilities for being an examinee 1 − *q_i_*.

The regression analysis was performed using R, version 3.4.3 (R Foundation for Statistical Computing, Vienna, Austria).^16^ Maps were illustrated with MANDARA, version 9.45.^17^

## RESULTS

Table 1 shows a summary of the population from the baseline survey of TUE in the dataset compared to that reported in the FHMS Official Report. The difference in the target populations between our dataset and the FHMS Official Report (367,649 − 359,200 = 8,449 in the primary evaluation and 2,293 − 2,246 = 47 in the confirmatory testing) reflected the number of individuals whose residence on March 11, 2011 was recorded as being outside Fukushima Prefecture or missing. The dataset in our analysis included 64,117 non-examinees (35,120 boys and 28,997 girls) for the primary evaluation and 194 non-examinees (68 boys and 126 girls) for the confirmatory testing. Thus, the percentage of non-examinees was 17.8% (19.1% for boys, 16.5% for girls) for the primary evaluation and 8.6% (9.0% for boys, 8.5% for girls) for the confirmatory testing.

**Table 1.** Summary of dataset from the baseline survey of thyroid examination of the Fukushima Health Management Survey (FHMS)

		Dataset in our analysis^a^		FHMS Official Report^b^	

		Examinees	Non-examinees^c^	Examinees	Non-examinees
**Primary evaluation**					
target population		359,200		367,649	
	
		295,083 (100%)	64,117 (100%)	300,473 (100%)	67,176 (100%)
	
Sex:	boys	148,814 (50.4%)	35,120 (54.8%)	151,683 (50.5%)	—
	girls	146,269 (49.6%)	28,997 (45.2%)	148,790 (49.5%)	—
Age:	0–5	85,803 (29.1%)	13,640 (21.3%)	87,794 (29.2%)	14,643 (21.8%)
	6–10	90,432 (30.6%)	3,698 (5.8%)	92,005 (30.6%)	3,985 (5.9%)
	11–15	84,854 (28.8%)	17,203 (26.8%)	86,120 (28.7%)	17,535 (26.1%)
	16–	33,994 (11.5%)	29,576 (46.1%)	34,554 (11.5%)	31,013 (46.2%)
Moving:	staying	246,912 (83.7%)	42,849 (66.8%)	—	—
	moved	42,477 (14.4%)	13,176 (20.5%)	—	—
	unknown	5,694 (1.9%)	8,092 (12.6%)	—	—
Area (i):	Middle	208,533 (70.7%)	35,463 (55.3%)	213,560 (71.1%)	37,978 (56.5%)
	Lowest	82,382 (27.9%)	28,115 (43.8%)	82,721 (27.5%)	28,663 (42.7%)
	Highest	4,168 (1.4%)	539 (0.8%)	4,192 (1.4%)	535 (0.8%)
Area (ii):	Ken-poku	77,849 (26.4%)	9,532 (14.9%)	78,906 (26.3%)	10,082 (15.0%)
	Ken-chu	82,093 (27.8%)	15,898 (24.8%)	85,521 (28.5%)	17,644 (26.3%)
	Ken-nan	23,706 (8.0%)	4,165 (6.5%)	23,877 (7.9%)	4,249 (6.3%)
	Aizu	30,579 (10.4%)	14,324 (22.3%)	30,569 (10.2%)	14,710 (21.9%)
	Minami-aizu	3,136 (1.1%)	1,473 (2.3%)	3,151 (1.0%)	1,496 (2.2%)
	Sou-sou	28,579 (9.7%)	5,994 (9.3%)	29,019 (9.7%)	6,132 (9.1%)
	Iwaki	49,141 (16.7%)	12,731 (19.9%)	49,430 (16.5%)	12,863 (19.1%)

**Confirmatory testing**					
target population		2,246		2,293	
	
		2,052 (100%)	194 (100%)	2,130 (100%)	163 (100%)
(confirmed diagnosis recorded)		(2,052 (100%))		2,090 (98.1%)	
	
Sex:	boys	690 (33.6%)	68 (35.1%)	775	
	girls	1,362 (66.4%)	126 (64.9%)	1,518	
Age:	0–5	91 (4.4%)	4 (2.1%)	95 (4.5%)	3 (1.8%)
	6–10	317 (15.4%)	27 (13.9%)	331 (15.5%)	22 (13.5%)
	11–15	903 (44.0%)	67 (34.5%)	933 (43.8%)	58 (35.6%)
	16–	741 (36.1%)	96 (49.5%)	771 (36.2%)	80 (49.1%)
Moving:	staying	1,642 (80.0%)	140 (72.2%)	—	—
	moved	351 (17.1%)	40 (20.6%)	—	—
	unknown	59 (2.9%)	14 (7.2%)	—	—
Area (i):	Middle	1,310 (63.8%)	126 (64.9%)	1,372 (64.4%)	107 (65.6%)
	Lowest	712 (34.7%)	66 (34.0%)	728 (34.2%)	54 (33.1%)
	Highest	30 (1.5%)	2 (1.0%)	30 (1.4%)	2 (1.2%)
Area (ii):	Ken-poku	429 (20.9%)	30 (15.5%)	441 (20.7%)	22 (13.5%)
	Ken-chu	597 (27.7%)	67 (34.5%)	641 (30.1%)	60 (36.8%)
	Ken-nan	137 (6.7%)	16 (8.2%)	143 (6.7%)	12 (7.4%)
	Aizu	270 (13.2%)	30 (15.5%)	273 (12.8%)	26 (16.0%)
	Minami-aizu	30 (1.5%)	5 (2.6%)	32 (1.5%)	3 (1.8%)
	Sou-sou	169 (8.2%)	16 (8.2%)	170 (8.0%)	15 (9.2%)
	Iwaki	420 (20.5%)	30 (15.5%)	430 (20.2%)	25 (15.3%)
					
**confirmed or suspected thyroid cancer cases**		115		116	
	boys/girls	39/76		39/77	

^a^Individuals whose residence were within Fukushima prefecture on March 11, 2011.

^b^Including individuals whose residence on March 11, 2011 were outside Fukushima prefecture or missing.

^c^Including individuals who did not agree to provide their information of the examination.

**Figure 2.**
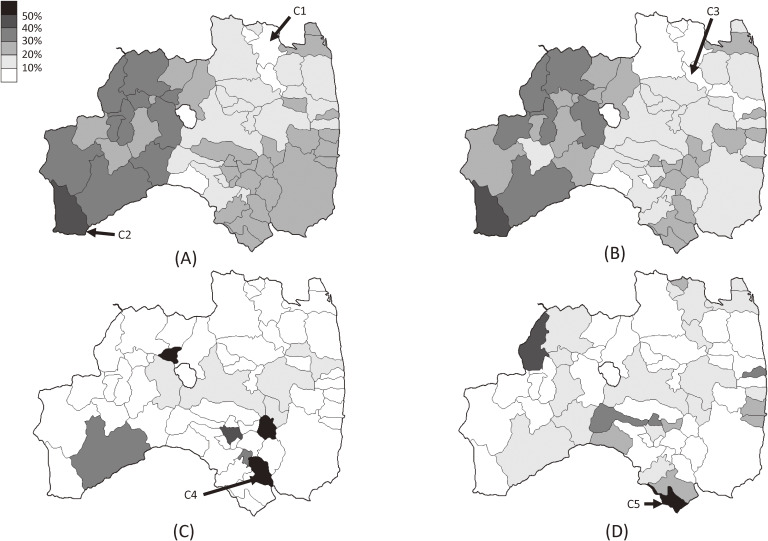
Maps of the proportion of non-examinees among the target population for each municipality including municipalities C1–C5: (A) boys and (B) girls in the primary evaluation, and (C) boys and (D) girls in the confirmatory testing.

Figure 2 shows the proportion of non-examinees among the target population in each municipality, based on sex: boys (Figure 2A, primary evaluation and Figure 2C, confirmatory testing) and girls (Figure 2B, primary evaluation and Figure 2D, confirmatory testing). First, in the primary evaluation, while the minimum and maximum values were 463/5,672 = 8.2% (C1 in Ken-poku) and 22/54 = 40.7% (C2 in Minami-aizu), respectively for boys, these values were 62/1,158 = 5.4% (C3 in Ken-poku) and 22/52 = 42.3% (C2 in Minami-aizu) for girls. For both sexes, the western parts of the prefecture—Aizu and Minami-aizu—showed higher proportions of non-examinees, while Ken-poku area tended to have lower proportions of non-examinees. Second, there were eight municipalities for boys and four municipalities for girls without target population for the confirmatory testing, and 34 and 18 municipalities for boys and girls, respectively, with 0% of non-examinees. The maximum values were 2/2 = 100% (C4 in Ken-nan) for boys and 1/2 = 50% (C5 in Ken-nan) for girls.

According to the cluster detection test, three areas were detected as significantly clustered areas (*P* < 0.05) in the primary evaluation for both sexes, as they were quite similar to each other (eFigure 2). On the other hand, no significantly clustered areas were identified in the confirmatory testing.

In order to evaluate associations between the proportion of non-examinees and demographic characteristics of the participants, we estimated the OR for each characteristic using multivariate logistic regression models. Table 2 shows the results of the primary evaluation of 359,200 individuals based on two models categorizing areas based on (i) exposure to radiation and (ii) administrative districts. The values of AIC were 276,245 for model (i) and 272,231 for model (ii), and those of the area under the receiver operating characteristic (ROC) curve (AUC), were calculated as 0.79 (i) and 0.80 (ii). The proportion of non-examinees among boys 0–5 years old who were classified as staying in the middle dose area and in Ken-poku was estimated as 9.9% and 7.4%, respectively, because the OR for the intercepts of the respective areas were 0.11 (i) and 0.08 (ii). Both models revealed that girls were not more likely to be non-examinees than boys (OR 0.80; 95% CI, 0.79–0.82 in model i and 0.78–0.81 in model ii), and among children 6–10 years old compared to those 0–5 years old (OR 0.27; 95% CI, 0.26–0.28 and OR 0.26; 95% CI, 0.25–0.27). However, children 11–15 years old and over 16 years old were likely to be non-examinees with ORs of 1.28 and over 5.00, respectively.

**Table 2.** Results showing the proportion of non-examinees for the primary evaluation based on two logistic regression models using areas based on (i) exposure to radiation and (ii) the administrative districts

Multivariate logistic regression model for Area (i)			

	Adjusted OR	(95% CI)	*P*

Intercept:	0.11		<0.001
Sex:			
boys	1.00		Ref.
girls	0.80	(0.79, 0.82)	<0.001
Age:			
0–5	1.00		Ref.
6–10	0.27	(0.26, 0.28)	<0.001
11–15	1.28	(1.25, 1.31)	<0.001
16–	5.11	(4.98, 5.24)	<0.001
Moving:			
staying (S)	1.00		Ref.
moved (M)	1.67	(1.62, 1.71)	<0.001
unknown (U)	4.66	(4.44, 4.89)	<0.001
Area:			
Middle (A1)	1.00		Ref.
Lowest (A2)	2.27	(2.22, 2.31)	<0.001
Highest (A3)	0.50	(0.45, 0.55)	<0.001
Interactions:			
M × A1	—		—
M × A2	—		—
M × A3	—		—
U × A1	—		—
U × A2	1.35	(1.24, 1.47)	<0.001
U × A3	—		—



Multivariate logistic regression model for Area (ii)			

	Adjusted OR	(95% CI)	*P*

Intercept:	0.08		<0.001
Sex:			
boys	1.00		Ref.
girls	0.80	(0.78, 0.81)	<0.001
Age:			
0–5	1.00		Ref.
6–10	0.26	(0.25, 0.27)	<0.001
11–15	1.28	(1.25, 1.31)	<0.001
16–	5.29	(5.16, 5.43)	<0.001
Moving:			
staying (S)	1.00		Ref.
moved (M)	1.72	(1.64, 1.79)	<0.001
unknown (U)	4.09	(3.81, 4.38)	<0.001
Area:			
Ken-poku (B1)	1.00		Ref.
Ken-chu (B2)	1.74	(1.68, 1.79)	<0.001
Ken-nan (B3)	1.59	(1.52, 1.66)	<0.001
Aizu (B4)	5.07	(4.89, 5.25)	<0.001
Minami-aizu (B5)	4.44	(4.11, 4.79)	<0.001
Sou-sou (B6)	1.86	(1.76, 1.97)	<0.001
Iwaki (B7)	2.29	(2.22, 2.37)	<0.001
Interactions:			
M × B1	—		—
M × B2	0.91	(0.85, 0.98)	0.009
M × B3	0.87	(0.79, 0.97)	0.012
M × B4	0.81	(0.75, 0.88)	<0.001
M × B5	—		—
M × B6	0.71	(0.66, 0.77)	<0.001
M × B7	—		—
U × B1	—		—
U × B2	1.50	(1.35, 1.67)	<0.001
U × B3	—		—
U × B4	1.16	(1.01, 1.34)	0.037
U × B5	2.00	(1.34, 2.98)	<0.001
U × B6	0.86	(0.74, 1.00)	0.047
U × B7	1.98	(1.77, 2.22)	<0.001


CI, confidence interval; OR, odds ratio.

For the area (i), a significant interaction was detected between the lowest dose area and unknown moving, indicating that the OR of unknown moving in the lowest dose area was slightly different from those in the middle and highest dose areas. However, these results showed that the OR to be a non-examinee was higher among 55,653 moved and 13,786 unknown moving individuals compared to those staying in the region. The lowest dose area had a higher OR and the highest dose area had a lower OR when compared to the middle dose area, implying that the lowest dose area (including Aizu, Minami-aizu, and Iwaki), showed 2.27/0.50 = 4.54 times higher tendency for the presence of a non-examinee compared to the highest dose area.

For the area (ii), nine interaction terms were determined to be significant. These results also showed that the moved and unknown moving groups had higher prevalence of non-examinees than the staying population in each area. Ken-poku area had the lowest incidence of non-examinees, whereas Aizu, Minami-aizu, and Iwaki had a higher incidence regardless of the moving status. In particular, comparison between the staying individuals showed that Aizu and Minami-aizu had very high ORs (OR 5.07; 95% CI, 4.89–5.25 and OR 4.44; 95% CI, 4.11–4.79, respectively), among the non-examinees compared to Ken-poku.

Table 3 summarizes the proportion of non-examinees following the confirmatory testing for 2,246 individuals. In this analysis, no interaction terms were found to be significant using either models (i) or (ii). The values of AICs were 1,315.4 (i) and 1,312.8 (ii), and those of AUCs were calculated as 0.57 (i) and 0.62 (ii). The proportion of the non-examinees among boys 0–5 years old who were classified as staying in the middle dose area and Ken-poku was estimated to be 3.8% and 2.9%, respectively. The ORs for almost all the factors showed a similar tendency to that observed in the primary evaluation (shown in Table 2). However, the OR for children 6–10 years old was over 1 compared to that observed for children 0–5 years old. Furthermore, most factors did not show any significant association with the proportion of non-examinees. Factors, such as age over 16 years old, unknown moving, and areas of Ken-chu and Aizu showed significant associations with the proportion of non-examinees compared with each reference category.

**Table 3.** Results showing the proportion of non-examinees for the confirmatory testing based on two logistic regression models using areas based on (i) exposure to radiation and (ii) the administrative districts

Multivariate logistic regression model for Area (i)			

	Adjusted OR	(95% CI)	*P*

Intercept:	0.04		<0.001
Sex:			
boys	1.00		Ref.
girls	0.95	(0.70, 1.31)	0.772
Age:			
0–5	1.00		Ref.
6–10	2.05	(0.70, 6.04)	0.192
11–15	1.74	(0.62, 4.90)	0.295
16–	2.88	(1.03, 8.05)	0.043
Moving:			
staying (S)	1.00		Ref.
moved (M)	1.29	(0.88, 1.90)	0.192
unknown (U)	2.49	(1.34, 4.62)	0.004
Area:			
Middle (A1)	1.00		Ref.
Lowest (A2)	1.04	(0.76, 1.42)	0.826
Highest (A3)	0.54	(0.12, 2.37)	0.417



Multivariate logistic regression model for Area (ii)			

	Adjusted OR	(95% CI)	*P*

Intercept:	0.03		<0.001
Sex:			
boys	1.00		Ref.
girls	0.94	(0.68, 1.28)	0.686
Age:			
0–5	1.00		Ref.
6–10	1.96	(0.66, 5.79)	0.222
11–15	1.74	(0.62, 4.91)	0.295
16–	3.03	(1.08, 8.47)	0.035
Moving:			
staying (S)	1.00		Ref.
moved (M)	1.24	(0.83, 1.84)	0.290
unknown (U)	2.43	(1.30, 4.52)	0.005
Area:			
Ken-poku (B1)	1.00		Ref.
Ken-chu (B2)	1.74	(1.11, 2.74)	0.016
Ken-nan (B3)	1.84	(0.97, 3.50)	0.063
Aizu (B4)	1.83	(1.07, 3.13)	0.028
Minami-aizu (B5)	2.78	(0.98, 7.82)	0.054
Sou-sou (B6)	1.31	(0.68, 2.52)	0.426
Iwaki (B7)	1.14	(0.67, 1.93)	0.635


CI, confidence interval; OR, odds ratio.

**Figure 3.**
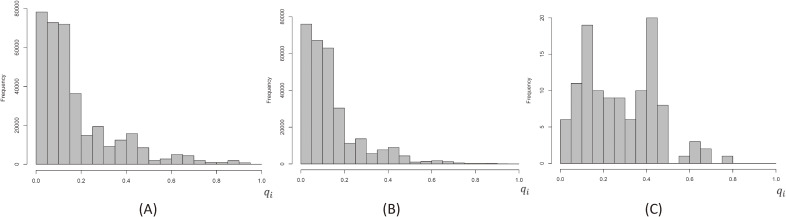
Histogram of predicted probabilities of non-examinees in the primary evaluation, (A) all target population, (B) all examinees, and (C) confirmed or suspected thyroid cancer cases.

Based on the logistic regression model for the area (ii) in the primary evaluation (Table 2), we predicted the probabilities *q_i_* for being a non-examinee for each individual *i*. Figure 3 shows the histogram of *q_i_* for (a) all target population (*n* = 359,200), (b) all examinees (*n* = 295,083), and (c) confirmed or suspected thyroid cancer cases (*n* = 115). The values of *q_i_* for 25^th^ percentile, median, and 75^th^ percentile were 0.066, 0.120, and 0.245, respectively, for (a); 0.044, 0.109, and 0.155, respectively, for (b); and 0.124, 0.285, and 0.414, respectively, for (c). These results showed that the confirmed or suspected thyroid cancer cases emerged from, not only populations with lower proportion, but also those with a higher proportion of non-examinees. A crude estimation of proportion of thyroid cancer cases was derived as 115/295,083 = 3.90 × 10^−4^ based upon the number of examinees in the primary evaluation. On the other hand, based upon the *q_i_* for non-examinees in the primary evaluation, the proportion was estimated with weights of 1/(1 − *q_i_*) as $\left(\sum_{i:\text{ thyroid cancer cases}}\frac{1}{1-q_{i}}\right)/\left(\sum_{i:\text{ examinee}}\frac{1}{1-q_{i}}\right)=4.78\times 10^{-4}$, then a naive estimation of the number of cases in the total target population was calculated as 171.7, where $\sum_{i:\text{ examinee}}\frac{1}{1-q_{i}}=\text{360,332.8}$.

## DISCUSSION

We assessed the proportion of non-examinees in the baseline survey of TUE conducted for the FHMS and found that it was associated with the demographic and personal characteristics (age, sex, residence on March 11, 2011, and moving after the accident) of 359,200 children and adolescents.

With regards to sex as a factor in the primary evaluation, it was indicated that girls were not more likely to be non-examinees than boys (OR 0.80), which implied a higher and statistically significant participation rate (OR 1.25; 95% CI, 1.23–1.28 for area ii). It was also shown that OR for girls was slightly lower than 1 in the confirmatory testing, although it was not statistically significant. One of the underlying reasons for this result might be the prior knowledge regarding the difference in the general incidence rates of thyroid cancer between the two sexes. The Center for Cancer Control and Information Services, National Cancer Center, Japan, has reported that the incidence ratios of thyroid cancer were higher for women than for men in Japan. If parents or students knew that females are more likely to develop thyroid cancer in general, they would become more cautious for their health, and might wish to feel safe by receiving a negative result that they were non-cases. It has been also found in the FHMS that females are more anxious and more likely to feel the effects of radiation stronger than males,^18^ which might have been contributed to the higher participation rate for girls.

After evaluating the age at the time of the earthquake as a factor, we found that the children 6–10 years old showed the lowest tendency to be non-examinees. The ORs were higher for children 11–15 years old and over 16 years old compared to those 0–5 years old. These results could be explained by the schooling status. Most children 6–10 years old received the evaluation when they were in elementary school. Typically, an elementary school belongs to a school district which is a small area, and it is believed that an evaluation by the school unit is easy to perform. However, the participation of children in the evaluation is dependent largely on their parents’ decisions, rather than their own. On the other hand, most children 11–15 years old and over 16 years old received the evaluation when they were in junior high or in high school. Their school districts were wider areas compared to those of the elementary schools, and units of local residential areas are not well defined. Furthermore, the participation of these older children in the evaluation is more dependent on their own intentions compared to that in elementary school children.

Based on the area of residence in the primary evaluation, as described in the results, there were significant interactions between the areas and moving status, but tendencies for non-examinees between areas and categories of moving did not change. The area in this dataset involves not only the geographical coordinates but also other factors, such as ecological and environmental characteristics, and socio-economic status (SES) for each area. In this evaluation, the area of residence on March 11, 2011 also determined the level of exposure to radiation caused by the Fukushima Daiichi Nuclear Power Plant accident, and the periods of examination. Detailed radiation maps were published by the Ministry of Education, Culture, Sports, Science, and Technology in 2011.^19^ The maps showed that radiation spread northwest from the accident site. It also showed that the west part of the prefecture, including Aizu, Minami-aizu, and the southern area of the plant including Iwaki were exposed to lower levels of radiation. Additionally, the municipalities chosen for the primary screening were allocated by fiscal years: 2011 for the evacuation zone comprising 13 municipalities near the nuclear power plant (10 in Sou-sou, 2 in Ken-poku and 1 in Ken-chu), 2012 for 12 municipalities in the middle part of the prefecture (6 in Ken-poku, 3 in Ken-chu, 3 in Ken-nan), and 2013 for the remaining regions, including the most inland part, Aizu, Minami-aizu, and coastal regions with Iwaki, except for the evacuation zone (34 municipalities). This phasing out of the evaluation probably had a great influence on the behavior of people participating in the survey. People in the areas with lower exposure to radiation, who were examined later, such as Aizu, Minami-aizu, and Iwaki, may not have been as enthusiastic about the evaluation as people in the areas that were examined earlier. As pointed out in previous studies,^5^^,^^10^ due to the geographical tendency of non-participation in the TUE, a cluster of high prevalence rates could be more likely to be detected in more contaminated areas. To assess the influence of non-examinees on regional prevalence rates of thyroid cancer, further detailed investigations may be required, considering adjustment for possible factors, including those found in this study.

Regarding the moving status, higher ORs were observed for non-examinees among people who moved compared with those who did not (staying). The moving status, in general, could be associated with the SES, and higher areal SES reflects better access to healthcare, leading to higher detection rates of the disease.^10^ However, in our study, people who moved after the accident may have missed the chance of being evaluated, depending on the area of their new residence. It is also possible that some individuals may have received other medical evaluation without attending the FHMS. Furthermore, our results suggested that there was no interaction for the proportion of non-examinees between change of residence and radiation exposure level. On the other hand, there were 13,786 individuals (5,694 examinees and 8,092 non-examinees) with unknown moving status during the primary evaluation. We could not clarify why and when their moving status became unknown. It is necessary to review these people in detail in our future studies. Furthermore, it is very important to follow these people in the survey of TUE.

Our employed model estimated the number of confirmed or suspected cases of thyroid cancer as 171.7 in the baseline survey, assuming that all the target population participated in the primary examination. However, the estimation did not consider the participation rate in the confirmatory testing phase, since it depended on the results of the primary examination. This estimate was higher than another naive estimate of 116/0.817 = 142 calculated from the reported 81.7% participation rate in the primary evaluation. Takahashi et al^8^ derived expected diagnosed cases with several sensitivity values using a cancer-progression model. Their model considered the participation rates by age and sex for the primary evaluation and confirmatory testing, and they simulated the number of cases to be 190.4 (49.3 for boys and 141.1 for girls) without loss of sensitivity. Our results suggest that it would be possible to acquire a more accurate estimation of the rates based not only on age and sex, but also on the area of residence and moving status. On the other hand, the sensitivity of testing was still an important factor for the estimation and requires further detailed research.

Our study has limitations. The moving status was not the same throughout the study, because the current residence is updated daily in the database. Therefore, we used the status as of October 25, 2017 for our analysis. However, because most people remained at their original residence, there was probably minimal impact on our results. Furthermore, our results imply that the proportion of non-examinees is not dependent only on their basic characteristics but also on confounding factors, such as social and cultural activities, which were not included in the present analysis. A more detailed study that includes these factors is required.

Despite these limitations, it was suggested that some demographic characteristics and change of residence may be influencing the proportion of non-examinees. Our results will help with the interpretation of reports and management of the FHMS. In particular, the first survey was conducted to establish the baseline and is, therefore, the most important for the subsequent evaluation programs in Fukushima. On the other hand, as data of the first and the second full-scale surveys are now being added to the database, participation rates for their examinations are also important to make accurate conclusions, which will be investigated in our future research. Furthermore, this accumulation of data will enable more detailed epidemiological studies in the future. Our findings and their applications will also be useful in future research regarding the necessity of performing ultrasound examinations in suspected cases of thyroid cancer.

## APPENDIX A. SUPPLEMENTARY DATA

eAppendix 1. Cluster detection test for the presence of non-examinees

eFigure 1. Flowchart of dataset generation

eFigure 2. Detected significant clustered areas of non-examinees in the primary evaluation: ① primary cluster, ② secondary cluster, and ③ tertiary cluster, for (A) boys and (B) girls.
