# Supplementary material for: Factors Influencing the Proportion of Non-examinees in the Fukushima Health Management Survey for Childhood and Adolescent Thyroid Cancer: Results From the Baseline Survey
Source: J Epidemiol. 2020 Jul 5;30(7):301–8. doi: 10.2188/jea.JE20180247 (PMC7280055; doi:10.2188/jea.JE20180247)
Supplement: Supplementary file 1 [file je-30-301-s001.pdf]

eAppendix 1. Cluster detection test for the presence of non-examinees

We applied a cluster detection test to investigate a regional tendency among the non-examinees, which allowed us to determine if their proportion pattern was completely random over the space of study without prior information, while indicating regions with a high non-examinee proportion. We employed the flexible scan statistic[13] implemented with the restricted likelihood ratio[14] under a binomial model to examine whether the non-participation rate showed any particular clustering when adjusted for the target population for each sex in each municipality. This allowed us to detect clusters of non-examinees under a statistical hypothesis test setting without undertaking multiple testing. We set two default arguments for the program: the maximum regional length of a cluster was set to 58 municipalities (total number of municipalities in the Fukushima Prefecture minus one), and the pre-specified significance level for a restriction was set as α_1=0.2.[14] The significance level of the test was set at α=0.05, and its *p*-value was calculated from 9,999 replications of the Monte Carlo hypothesis testing. The cluster detection test was performed using FleXScan, version 3.1.2.[20]

Flexible scan statistic detected three areas as significantly clustered in the primary evaluation for both sexes, as they were quite similar to each other (**eFigure 2A**, boys and **eFigure 2B**, girls). All these clusters had statistically significant *p*-values $p<0.001$. For boys, the primary clustered area with 16 municipalities in the west part of the prefecture had 8,246 non-examinees out of 24,925 individuals; the second clustered area with 17 municipalities had 11,723 non-examinees out of 51,666 individuals, and the third clustered area with one municipality had 839 non-examinees out of 3,350 individuals. For girls, the primary clustered area included the same 16 municipalities that formed the primary clustered area for boys. However, one municipality was not included, whereas another municipality was included therein. Out of 24,184 individuals, 7,459 non-examinees were observed. The second clustered area with 18 municipalities included the same 17 areas that formed the second clustered area for boys plus one municipality. Out of 49,365 individuals, 9,539 non-examinees were identified. The third clustered area was the same as that for the boys and had 665 non-examinees out of 3,269 individuals. These results revealed that the west part of the prefecture showed a higher proportion of non-examinees. Moreover, the area around Iwaki also had a higher proportion of non-examinees in the primary evaluation.

On the other hand, there were no significantly clustered areas in the confirmatory testing.

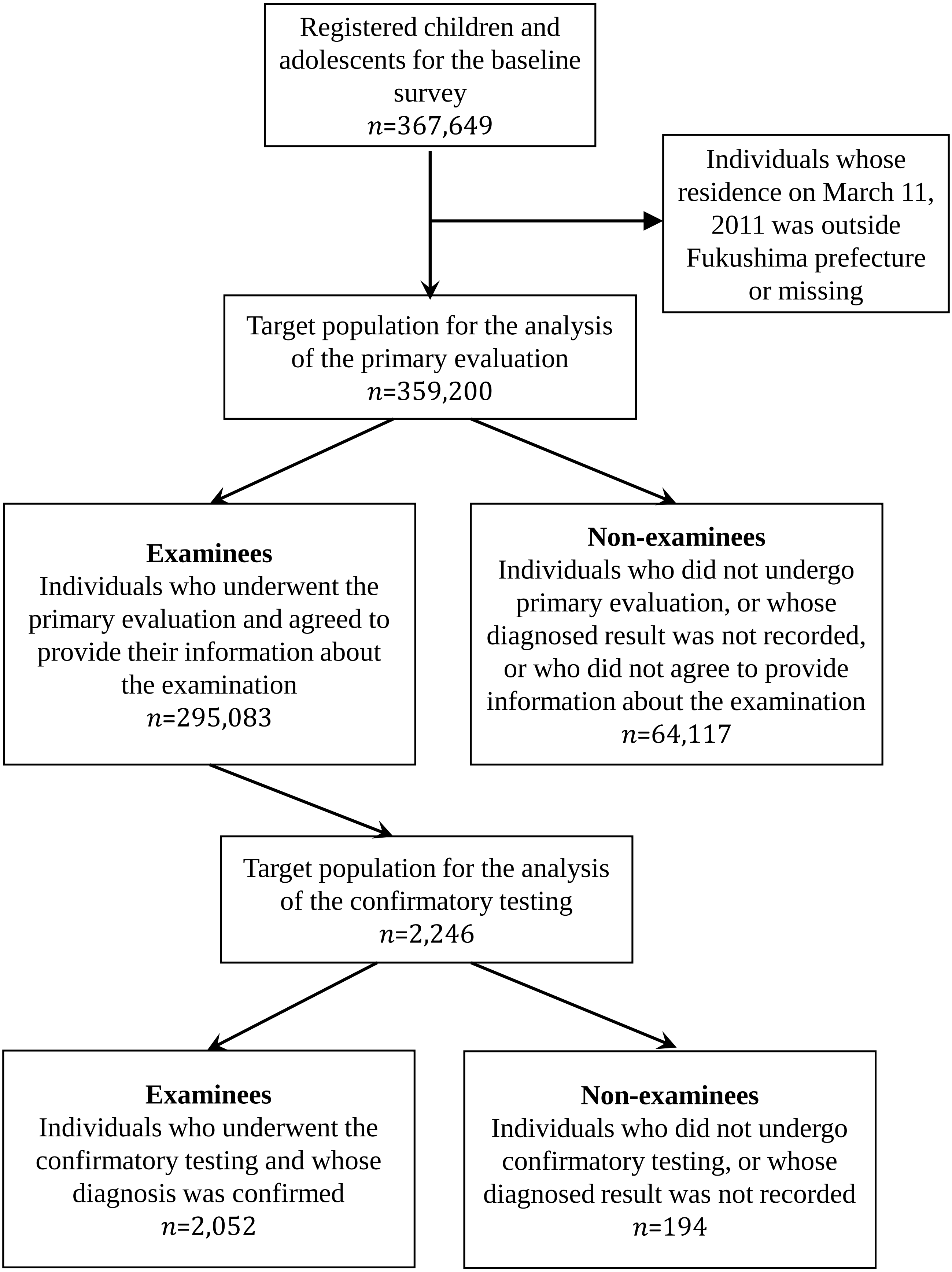

eFigure 1. Flowchart of dataset generation

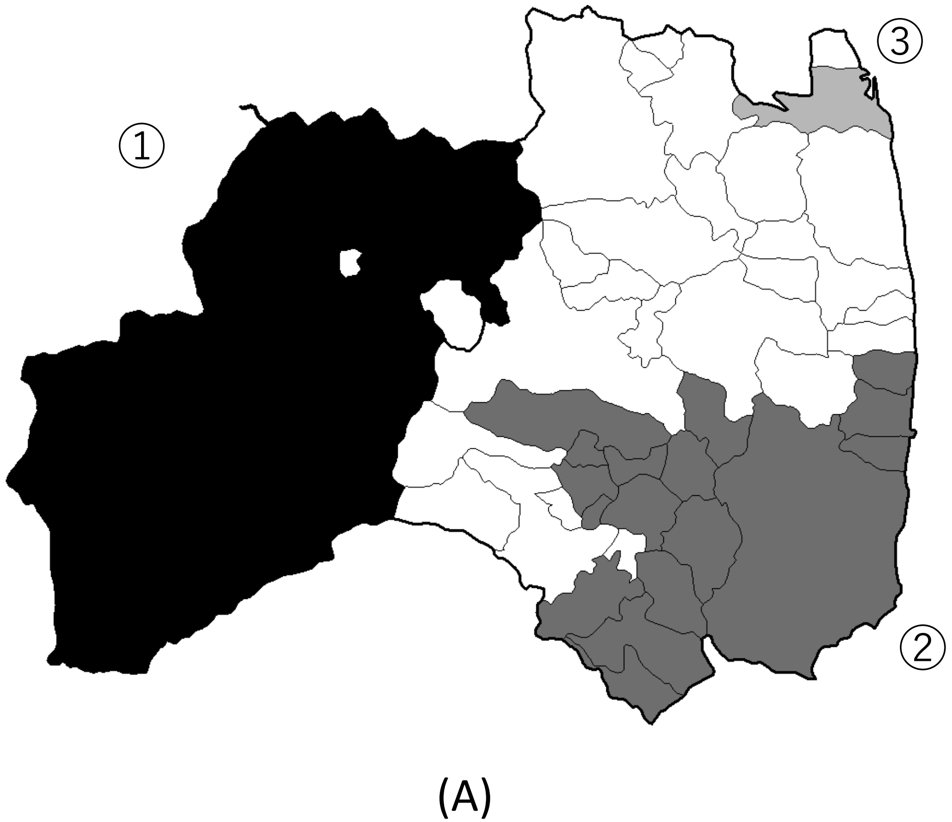

eFigure 2. Detected significant clustered areas of non-examinees in the primary evaluation:
① primary cluster, ② secondary cluster, and ③ tertiary cluster, for (A) boys and (B) girls.
